# Polar Transversal Initial Alignment Algorithm for UUV with a Large Misalignment Angle

**DOI:** 10.3390/s18103231

**Published:** 2018-09-25

**Authors:** Zheping Yan, Lu Wang, Tongda Wang, Honghan Zhang, Zewen Yang

**Affiliations:** 1 College of Automation, Harbin Engineering University, Harbin 150001, China; yanzheping@hrbeu.edu.cn (Z.Y.); yangzewen@hrbeu.edu.cn (Z.Y.); 2 Beijing Mechanical & Electrical Overall Design Department, The Fourth Academy of China Aerospace Science & Industry Corporation (CASIC), Beijing 100854, China; ins_wtd@163.com

**Keywords:** Strapdown Inertial Navigation System, polar transversal navigation, initial alignment algorithm, large misalignment angle, simplified unscented Kalman filter

## Abstract

The conventional initial alignment algorithms are invalid in the polar region. This is caused by the rapid convergence of the Earth meridians in the high-latitude areas. However, the initial alignment algorithms are important for the accurate navigation of Unmanned Underwater Vehicles. The polar transversal initial alignment algorithm is proposed to overcome this problem. In the polar transversal initial alignment algorithm, the transversal geographic frame is chosen as the navigation frame. The polar region in the conventional frames is equivalent to the equatorial region in the transversal frames. Therefore, the polar transversal initial can be effectively applied in the polar region. According to the complex environment in the polar region, a large misalignment angle is considered in this paper. Based on the large misalignment angle condition, the non-linear dynamics models are established. In addition, the simplified unscented Kalman filter (UKF) is chosen to realize the data fusion. Two comparison simulations and an experiment are performed to verify the performance of the proposed algorithm. The simulation and experiment results indicate the validity of the proposed algorithm, especially when large misalignment angles occur.

## 1. Introduction

Unmanned Underwater Vehicles (UUVs) are important equipment for marine exploration and investigation. Strapdown Inertial Navigation Systems (SINSs) have the advantages of autonomy and concealment [1]. Therefore, SINSs are widely used in the navigation of UUVs. SINS is a process that continuously infers the navigation states based on the initial conditions and the results of the inertial measurement unit (IMU). Therefore, the initial conditions are very important for the SINS. The initial alignment algorithm is an important way to provide the accurate initial conditions for the SINS. Scholars have conducted a lot of research on initial alignment algorithms for the non-polar region. In addition, initial alignment algorithms for the non-polar region are relatively mature [2,3,4,5]. However, the polar environment is very special. In the high-latitude areas, the Earth meridians converge at the poles quickly. This creates certain challenges for the navigation of UUVs in the polar region [6,7,8]. Many of the relatively mature initial alignment algorithms applied in the non-polar region cannot be applied in the polar region normally because of the quick convergence of the Earth’s meridians. The initial alignment algorithms in the polar region are still in their infancy [9,10]. The transversal coordinate system is obtained by rotating the conventional latitude and longitude coordinate system by 90° around the y axis of the earth-centered-earth fixed frame. The proposed transversal coordinate system can well solve the problem that conventional algorithms cannot be applied normally in the polar region. At the same time, due to the complex environment in the polar region, large misalignment angles need to be considered in UUVs. Therefore, a polar transversal initial alignment algorithm for UUVs with a large misalignment angle is proposed for the requirements of polar UUV initial alignment.

The harsh natural environment in the polar region creates certain difficulties to the application of conventional non-polar navigation and the initial alignment algorithms in the polar region. For example, the presence of polar multipath effects and tropospheric delay makes the Global Navigation Satellite System (GNSS) invalid in the polar region [11]. The abnormality of the geomagnetic field near the polar region makes it impossible to use the geomagnetic navigation method in the polar region. Due to the rapid convergence of the Earth meridians and the latitude approaching 90°, the initial alignment algorithm based on the conventional north-oriented SINS cannot be applied normally in the polar region. In the conventional north-oriented mechanical arrangement, the up angular component errors of the commanded angular velocity is tangential to the latitude. Therefore, when the latitude approaches 90°, the error approaches infinity. Initial alignment algorithms based on the north-oriented SINS have problems such as calculation overflow and error amplification when applied in the polar region [12]. Therefore, initial alignment algorithms based on north-oriented SINS are invalid in the polar region. The definition model of the conventional latitude and longitude causes this problem. Therefore, changing the latitude and longitude definition model can solve this problem. By rotating the conventional latitude and longitude coordinate system by 90° around the y axis of the earth-centered earth-fixed frame, a transversal latitude and longitude coordinate system can be obtained. Based on the definition model of the transversal latitude and longitude coordinate system, a series of transversal frames such as the transversal geographic frame and the transversal earth frame can be obtained. The points near the polar region in the conventional coordinate system correspond to the points near the equator in the transversal coordinate system. Therefore, the initial alignment algorithm based on the transversal coordinate system can be applied normally in the polar region. The concept of the transversal coordinate system was mentioned by a scholar in his monograph in 1964 [13]. However, it was only briefly mentioned. Some scholars have also mentioned the transversal coordinate system in their papers [14,15,16,17]. However, the detailed mechanical arrangement and the transversal initial alignment algorithms were not included in these papers. Therefore, to meet the requirements of polar initial alignment in the case of a large misalignment angle, in the following sections, this paper will deduce the polar transversal initial alignment algorithm for UUVs with a large misalignment angle. This algorithm is very important for the requirements of the polar initial alignment.

A lot of algorithms have been proposed for the initial alignment algorithms in the non-polar region [18,19,20,21]. According to the misalignment angle, the initial alignment algorithms can be divided into initial alignment algorithms suitable for small misalignment angles and initial alignment algorithms suitable for large misalignment angles. In the case of small misalignment angles, the established initial alignment dynamics model is a linear model. Therefore, the Kalman filter (KF) can be used to solve the problem of data fusion [22,23]. When the misalignment angle is large, the established initial alignment dynamics model is a nonlinear model. At this point, the KF is no longer applicable. In addition, non-linear filter algorithms should be selected to achieve the data fusion. The simplified unscented Kalman filter (UKF) is chosen to achieve the data fusion of the polar transversal initial alignment algorithm for UUVs with a large misalignment angle [24,25,26,27].

A polar transversal initial alignment algorithm for UUVs with a large misalignment angle is proposed in this paper. The main contributions of this paper include the following two aspects. First, considering the special environment in the polar region, the transversal initial alignment algorithm is deduced based on the transversal frames and the characteristics of UUV. This algorithm can overcome the problems caused by the rapid convergence of the Earth meridians in the high-latitude areas. Second, large misalignment angles of UUVs and nonlinear dynamics models are considered in this paper. A simplified UKF is chosen for the data fusion. The sections are arranged as follows. The transversal frames and the relationships among these frames are discussed in Section 2. The error equations of the polar transversal initial alignment algorithm for UUVs with large misalignment angles are deduced in Section 3. Based on Section 3, the dynamics model and the observation model are established in Section 4. The simplified UKF is also described in Section 4 to realize the nonlinear data fusion. The simulation and the experiments are performed in Section 5. In addition, the results are also discussed in Section 5. Finally, the conclusion is given in Section 6.

## 2. The Transversal Frames and the Relationship among the Frames

In the polar region, the Earth meridians converge quickly. In addition, the latitude approaches 90° in the polar region. These cause inaccuracy and calculation overflow in the conventional algorithms when they are applied in the polar region. Therefore, the conventional algorithm based on the earth-centered earth-fixed (ECEF) frame is invalid in the polar region. To solve these problems, transversal frames are proposed in this section. The polar transversal initial alignment algorithm is deduced based on the proposed transversal frames in the following sections. The detailed definitions of transversal frames and the relationships among the frames are described in the following text. To simplify the description, the frames related in this paper are expressed as follows: n frame represents the navigation frame; i frame represents the inertial frame; b frame represents the body frame of the UUV; e frame represents the earth-centered earth-fixed frame; g frame represents the geographic frame; $e_{t}$ frame represents the transversal earth-centered earth-fixed frame; $g_{t}$ frame represents the transversal geographic frame.

### 2.1. The Transversal Frames

**Figure 1:**
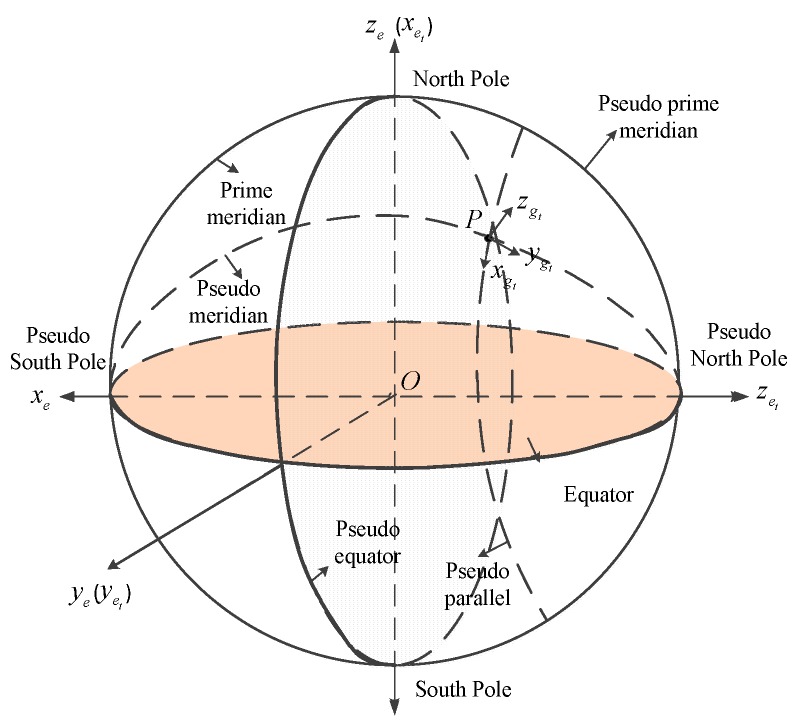
The definition of the transversal frames.

According to the analysis above, the reason causing the conventional algorithms to be invalid in the polar region is the rapid convergence of the Earth meridians in high-latitude areas. The conventional latitude and longitude are defined by people to facilitate the representation of position. The classic definition of latitude and longitude causes difficulties for conventional algorithms when applied in polar regions. Therefore, changing the definition of the classic latitude and longitude can solve the problem fundamentally. The definition of the transversal frames is shown in Figure 1. The South Pole, the North Pole, the equator, the prime meridian, the meridian, the parallel, the latitude and the longitude in the transversal latitude and longitude coordinate system are different from those in the conventional latitude and longitude coordinate system. Therefore, they are redefined as the pseudo South Pole, the pseudo North Pole, the pseudo equator, the pseudo prime meridian, the pseudo meridian, the pseudo parallel, the pseudo latitude and the pseudo longitude in the transversal latitude and longitude coordinate system. The redefined pseudo South Pole, the pseudo North Pole, the pseudo equator, the pseudo prime meridian, the pseudo meridian, the pseudo parallel, the pseudo latitude and the pseudo longitude are described in the following text.

In the transversal latitude and longitude coordinate system, the pseudo South Pole is the intersection of the equator and the 0° meridian. The pseudo North Pole is the intersection of the equator and the 180° meridian. The pseudo equator is the plane though the North Pole and the South Pole and perpendicular to the line connecting the pseudo North Pole and the pseudo South Pole. The pseudo prime meridian is the 0°/180° meridian coil which passes though the North Pole and the South Pole. The pseudo meridians are the lines on the surface of the earth connecting the pseudo North Pole and the pseudo South Pole. The pseudo parallels are the lines perpendicular to the pseudo meridian. The pseudo latitude is the line-surface angle formed by the line from the point connecting to the earth’s core and the pseudo equatorial plane. The pseudo longitude is the dihedral angle formed by the plane though the pseudo meridian of the point and the plane though the pseudo prime meridian.

Based on the definition of the transversal latitude and longitude coordinate system, the transversal earth-centered earth-fixed frame ($e_{t}$ frame) and the transversal geographic frame ($g_{t}$ frame) can be described as follows. The origin and the axes of the transversal earth-centered earth-fixed frame ($e_{t}$ frame) can be expressed as:

Origin (O)—the centroid of the earth; coincides with the origin of the e frame;
X axis ($x_{e_{t}}$)—the point at the intersection of the pseudo equator and the pseudo prime meridian;
Z axis ($z_{e_{t}}$)—the point at the pseudo North Pole;
Y axis ($y_{e_{t}}$)—the point on the pseudo equator, comprising a right-handed rectangular coordinate system with $x_{e_{t}}$ and $z_{e_{t}}$.

The origin and the axes of the transversal geographic frame ($g_{t}$ frame) can be expressed as:

Origin (P)—coincides with the position of the UUV;
X axis ($x_{g_{t}}$)—the point to the pseudo east;
Y axis ($y_{g_{t}}$)—the point to the pseudo north;
Z axis ($z_{g_{t}}$)—the point in the up direction; coincides with $z_{g}$.

### 2.2. Relationships among the Related Frames

According to the definition of the $e_{t}$ frame, the direction cosine matrix between the e frame and the $e_{t}$ frame can be expressed as Equation (1). The relationship between the conventional and the transversal latitude and longitude coordinate system can be expressed as Equations (2)–(5).

(1) $$C_{e}^{e_{t}}=\left[\begin{array}{ccc} \cos\left(-\frac{\pi }{2}\right) & 0 & -\sin\left(-\frac{\pi }{2}\right)\\ 0 & 1 & 0\\ \sin\left(-\frac{\pi }{2}\right) & 0 & \cos\left(-\frac{\pi }{2}\right) \end{array}\right]=\left[\begin{array}{ccc} 0 & 0 & 1\\ 0 & 1 & 0\\ -1 & 0 & 0 \end{array}\right],$$

(2) $$L_{t}=-\arcsin(\cos L\cos\lambda ),$$

(3) $$\lambda_{t}=\arctan(\cos L\sin\lambda /\sin L),$$

(4) $$L=\arcsin(\cos L_{t}\cos\lambda_{t}),$$

(5) $$\lambda =-\arctan(\cos L_{t}\sin\lambda_{t}/\sin L_{t}),$$

where L, λ and h are the longitude, the latitude and the height in the conventional latitude and longitude coordinate system, respectively. $L_{t}$, $\lambda_{t}$ and $h_{t}$ are those in the transversal coordinate system, respectively.

For other related frames, such as the e frame, the g frame, the $e_{t}$ frame and the $g_{t}$ frame, the relationships among them can be described as Equations (6)–(8).

(6) $$C_{e}^{g}=\left[\begin{array}{ccc} -\sin\lambda & \cos\lambda & 0\\ -\sin L\cos\lambda & -\sin L\sin\lambda & \cos L\\ \cos L\cos\lambda & \cos L\sin\lambda & \sin L \end{array}\right],$$

(7) $$C_{e_{t}}^{g_{t}}=\left[\begin{array}{ccc} -\sin\lambda_{t} & \cos\lambda_{t} & 0\\ -\sin L_{t}cos\lambda_{t} & -\sin L_{t}\sin\lambda_{t} & \cos L_{t}\\ \cos L_{t}\cos\lambda_{t} & \cos L_{t}\sin\lambda_{t} & \sin L_{t} \end{array}\right],$$

(8) $$C_{g}^{g_{t}}=C_{e_{t}}^{g_{t}}C_{e}^{e_{t}}C_{g}^{e},$$

## 3. Error Equations of the Polar Transversal Initial Alignment Algorithm for UUV

### 3.1. Attitude Error Equations of UUV

Considering the large misalignment angles of UUVs in the polar transversal initial alignment algorithm, there are errors between the ideal and the actual transversal geographic frames. The errors between the ideal and the actual transversal geographic frames can be described as $\phi^{g_{t}}={\left[\phi_{x}^{g_{t}}\;\phi_{y}^{g_{t}}\;\phi_{z}^{g_{t}}\right]}^{T}$. The direction cosine matrix $C_{g_{t}}^{g_{t}{}^{\prime }}$ from the $g_{t}$ frame to the $g_{t}{}^{\prime }$ frame can be described as Equation (9).

(9) $$C_{g_{t}}^{g_{t}{}^{\prime }}=\left[\begin{array}{ccc} c\phi_{y}^{g_{t}}c\phi_{z}^{g_{t}}-s\phi_{y}^{g_{t}}s\phi_{x}^{g_{t}}s\phi_{z}^{g_{t}} & c\phi_{y}^{g_{t}}s\phi_{z}^{g_{t}}+s\phi_{y}^{g_{t}}s\phi_{x}^{g_{t}}c\phi_{z}^{g_{t}} & -s\phi_{y}^{g_{t}}c\phi_{x}^{g_{t}}\\ -c\phi_{x}^{g_{t}}s\phi_{z}^{g_{t}} & c\phi_{x}^{g_{t}}c\phi_{z}^{g_{t}} & s\phi_{x}^{g_{t}}\\ s\phi_{y}^{g_{t}}c\phi_{z}^{g_{t}}+c\phi_{y}^{g_{t}}s\phi_{x}^{g_{t}}s\phi_{z}^{g_{t}} & s\phi_{y}^{g_{t}}s\phi_{z}^{g_{t}}-c\phi_{y}^{g_{t}}s\phi_{x}^{g_{t}}c\phi_{x}^{g_{t}} & c\phi_{y}^{g_{t}}c\phi_{x}^{g_{t}} \end{array}\right],$$

(10) $$\omega_{g_{t}g_{t}{}^{\prime }}^{g_{t}{}^{\prime }}=\left[\begin{array}{ccc} c\phi_{y}^{g_{t}} & 0 & -s\phi_{y}^{g_{t}}c\phi_{x}^{g_{t}}\\ 0 & 1 & s\phi_{x}^{g_{t}}\\ s\phi_{y}^{g_{t}} & 0 & c\phi_{y}^{g_{t}}c\phi_{x}^{g_{t}} \end{array}\right]\left[\begin{array}{c} {\dot{\phi }}_{x}^{g_{t}}\\ {\dot{\phi }}_{y}^{g_{t}}\\ {\dot{\phi }}_{z}^{g_{t}} \end{array}\right]=C_{t}{\dot{\phi }}^{g_{t}},$$

(11) $${\dot{\phi }}^{g_{t}}=C_{t}{}^{-1}\omega_{g_{t}g_{t}{}^{\prime }}^{g_{t}{}^{\prime }}=\frac{\omega_{g_{t}g_{t}{}^{\prime }}^{g_{t}{}^{\prime }}}{\cos\phi_{x}^{g_{t}}}\cdot \left[\begin{array}{ccc} \cos\phi_{y}^{g_{t}}\cos\phi_{x}^{g_{t}} & 0 & \sin\phi_{y}^{g_{t}}\cos\phi_{x}^{g_{t}}\\ \sin\phi_{y}^{g_{t}}\sin\phi_{x}^{g_{t}} & \cos\phi_{y}^{g_{t}} & -\cos\phi_{y}^{g_{t}}\sin\phi_{x}^{g_{t}}\\ -\sin\phi_{y}^{g_{t}} & 0 & \cos\phi_{y}^{g_{t}} \end{array}\right],$$

where s(⋅) and c(⋅) represent sin(⋅) and cos(⋅), respectively.

The attitude differential equations of the polar transversal initial alignment algorithm with a large misalignment angle in an ideal and an actual condition can be described as Equations (12) and (13), respectively.

(12) $${\dot{C}}_{b}^{g_{t}}=C_{b}^{g_{t}}\left[\omega_{ib}^{b}\times \right]-\left[\omega_{ig_{t}}^{g_{t}}\times \right]C_{b}^{g_{t}},$$

(13) $${\dot{C}}_{b}^{g_{t}{}^{\prime }}=C_{b}^{g_{t}{}^{\prime }}\left[{\hat{\omega }}_{ib}^{b}\times \right]-\left[{\hat{\omega }}_{ig_{t}}^{g_{t}}\times \right]C_{b}^{g_{t}{}^{\prime }},$$

where

(14) $${\hat{\omega }}_{ib}^{b}=\omega_{ib}^{b}+\delta \omega_{ib}^{b}=\omega_{ib}^{b}+\varepsilon^{b},$$

(15) $${\hat{\omega }}_{ig_{t}}^{g_{t}}={\hat{\omega }}_{ie}^{g_{t}}+{\hat{\omega }}_{eg_{t}}^{g_{t}},$$

(16) $${\hat{\omega }}_{ie}^{g_{t}}=\omega_{ie}^{g_{t}}+\delta \omega_{ie}^{g_{t}},$$

(17) $${\hat{\omega }}_{eg_{t}}^{g_{t}}=\omega_{eg_{t}}^{g_{t}}+\delta \omega_{eg_{t}}^{g_{t}},$$

where $\delta \omega_{ib}^{b}$, $\delta \omega_{ie}^{g_{t}}$ and $\delta \omega_{eg_{t}}^{g_{t}}$ represent the errors of $\omega_{ib}^{b}$, $\omega_{ie}^{g_{t}}$ and $\omega_{eg_{t}}^{g_{t}}$ in actual conditions, respectively. $\varepsilon^{b}$ is the gyro drift. $\varepsilon_{c}^{b}$ is the gyro constant drift. In addition, $\varepsilon_{w}^{b}$ is the gyro random drift. The velocity error of the polar transversal initial alignment algorithm can be described as $\delta V^{g_{t}}={\left[\delta v_{E_{t}}\;\delta v_{N_{t}}\;\delta v_{U_{t}}\right]}^{T}$. The UUV is supposed to be temporarily anchored. Therefore, to simplify the analysis and for the purposes of this paper, the position errors are assumed to be zero.

(18) $$\omega_{eg_{t}}^{g_{t}}=\omega_{ee_{t}}^{g_{t}}+\omega_{e_{t}g_{t}}^{g_{t}}=C_{e_{t}}^{g_{t}}\omega_{ee_{t}}^{e_{t}}+\omega_{e_{t}g_{t}}^{g_{t}}=\omega_{e_{t}g_{t}}^{g_{t}},$$

(19) $$\omega_{e_{t}g_{t}}^{g_{t}}=\left[\begin{array}{c} -v_{N_{t}}/R_{oth}\\ v_{E_{t}}/R_{oth}\\ \left(v_{E_{t}}\tan L_{t}\right)/R_{oth} \end{array}\right]=\left[\begin{array}{c} -{\dot{L}}_{t}\\ {\dot{\lambda }}_{t}\cos L_{t}\\ {\dot{\lambda }}_{t}\sin L_{t} \end{array}\right]$$

(20) $$\delta \omega_{eg_{t}}^{g_{t}}={\hat{\omega }}_{eg_{t}}^{g_{t}}-\omega_{eg_{t}}^{g_{t}}=\left[\begin{array}{ccc} 0 & -\frac{1}{R_{oth}} & 0\\ \frac{1}{R_{oth}} & 0 & 0\\ \frac{\tan L_{t}}{R_{oth}} & 0 & 0 \end{array}\right]\left[\begin{array}{c} \delta v_{E_{t}}\\ \delta v_{N_{t}}\\ \delta v_{U_{t}} \end{array}\right]=C_{\omega eg_{t}v}\delta V^{g_{t}},$$

(21) $$C_{\omega eg_{t}v}=\left[\begin{array}{ccc} 0 & -\frac{1}{R_{oth}} & 0\\ \frac{1}{R_{oth}} & 0 & 0\\ \frac{\tan L_{t}}{R_{oth}} & 0 & 0 \end{array}\right],$$

where

(22) $$R_{oth}=R_{ot}+h_{t},$$

(23) $$R_{Mt}=R_{e}\left(1-e^{2}\right)/{\left(1-e^{2}\sin^{2}L\right)}^{\frac{3}{2}}=R_{e}\left(1-e^{2}\right)/{\left(1-e^{2}\cos^{2}L_{t}\cos^{2}\lambda_{t}\right)}^{\frac{3}{2}},$$

(24) $$R_{Nt}=R_{e}/{\left(1-e^{2}\cos^{2}L_{t}\cos^{2}\lambda_{t}\right)}^{\frac{1}{2}},$$

(25) $$R_{ot}=\sqrt{R_{Mt}R_{Nt}},$$

Subtracting Equation (12) from (13), considering Equations (14)–(25), the attitude error equation of the polar transversal initial alignment algorithm can be expressed as Equation (26).

(26) $${\dot{\phi }}^{g_{t}}=C_{t}{}^{-1}\left[\left(I-C_{g_{t}}^{g_{t}{}^{\prime }}\right)\omega_{ig_{t}}^{g_{t}}+C_{\omega eg_{t}v}\delta V^{g_{t}}-C_{b}^{g_{t}{}^{\prime }}\varepsilon^{b}\right],$$

### 3.2. Velocity Error Equations for UUVs

The velocity differential equations of the polar transversal initial alignment under ideal conditions and under actual conditions can be expressed as Equations (27) and (28), respectively.

(27) $${\dot{V}}^{g_{t}}=f^{g_{t}}-\left(2\omega_{ie}^{g_{t}}+\omega_{eg_{t}}^{g_{t}}\right)\times V^{g_{t}}+g^{g_{t}},$$

(28) $${\dot{\hat{V}}}^{g_{t}}={\hat{f}}^{g_{t}}-\left(2{\hat{\omega }}_{ie}^{g_{t}}+{\hat{\omega }}_{eg_{t}}^{g_{t}}\right)\times {\hat{V}}^{g_{t}}+{\hat{g}}^{g_{t}},$$

where $f^{g_{t}}=C_{b}^{g_{t}}f^{b}$ and $f^{b}$ is the special force measured by IMU in the transversal SINS. $g^{g_{t}}=C_{b}^{g_{t}}g^{b}={\left[0\;0\;-g\right]}^{T}$. Under actual conditions, there are errors during updating. $\delta V^{g_{t}}$, $\nabla^{b}$, $\delta \omega_{ie}^{g_{t}}$, $\delta \omega_{eg_{t}}^{g_{t}}$ and $\delta g^{g_{t}}$ represent the errors of $V^{g_{t}}$, $f^{b}$, $\omega_{ie}^{G}$, $\omega_{eG}^{G}$ and $g^{G}$, respectively.

(29) $${\hat{V}}^{g_{t}}=V^{g_{t}}+\delta V^{g_{t}},$$

(30) $${\hat{C}}_{b}^{g_{t}}=C_{g_{t}}^{g_{t}{}^{\prime }}C_{b}^{g_{t}}=\left(I-\phi^{g_{t}}\times \right)C_{b}^{g_{t}},$$

(31) $${\hat{f}}^{b}=f^{b}+\nabla^{b},$$

(32) $$\nabla^{b}=\nabla_{c}^{b}+\nabla_{w}^{b},$$

(33) $${\hat{\omega }}_{ie}^{g_{t}}=\omega_{ie}^{g_{t}}+\delta \omega_{ie}^{g_{t}},$$

(34) $${\hat{\omega }}_{eg_{t}}^{g_{t}}=\omega_{eg_{t}}^{g_{t}}+\delta \omega_{eg_{t}}^{g_{t}},$$

(35) $${\hat{g}}^{g_{t}}=g^{g_{t}}+\delta g^{g_{t}},$$

where $\nabla^{b}$ is the accelerometer bias. $\nabla_{c}^{b}$ is the accelerometer constant bias. $\nabla_{w}^{b}$ is the accelerometer random bias.

Subtracting Equation (27) from (28), considering Equations (29)–(35), the velocity error equation can be expressed as Equation (36).

(36) $$\delta {\dot{V}}^{g_{t}}=\left[I-(C_{g_{t}}^{g_{t}{}^{\prime }}){}^{T}\right]C_{b}^{g_{t}}f^{b}+\left[V^{g_{t}}\times C_{\omega eg_{t}v}-\left(2\omega_{ie}^{g_{t}}+\omega_{eg_{t}}^{g_{t}}\right)\times \right]\cdot \delta V^{g_{t}}+C_{b}^{g_{t}{}^{\prime }}\nabla^{b},$$

## 4. Filter Models and Filter Algorithm

### 4.1. Dynamics Model

The states to be estimated in the polar transversal initial alignment algorithm are the attitude errors $\phi^{g_{t}}$, the velocity errors $\delta V^{g_{t}}$, the gyro drifts $\varepsilon_{c}^{b}$ and the accelerometer bias $\nabla_{c}^{b}$. Based on the analysis in Section 3, the dynamics model can be expressed as Equation (37).

(37) $$\{\begin{array}{l} {\dot{\phi }}^{g_{t}}=C_{t}{}^{-1}\left[\left(I-C_{g_{t}}^{g_{t}{}^{\prime }}\right)\omega_{ig_{t}}^{g_{t}}+C_{\omega eg_{t}v}\delta V^{g_{t}}-C_{b}^{g_{t}{}^{\prime }}\varepsilon^{b}\right]\\ \delta {\dot{V}}^{g_{t}}=\left[I-(C_{g_{t}}^{g_{t}{}^{\prime }}{)}^{T}\right]C_{b}^{g_{t}}f^{b}+\left[V^{g_{t}}\times C_{\omega eg_{t}v}-\left(2\omega_{ie}^{g_{t}}+\omega_{eg_{t}}^{g_{t}}\right)\times \right]\cdot \delta V^{g_{t}}+C_{b}^{g_{t}{}^{\prime }}\nabla^{b}\\ {\dot{\varepsilon }}^{b}=0\\ {\dot{\nabla }}^{b}=0 \end{array},$$

where $C_{t}{}^{-1}$ can be described as:

(38) $$C_{t}{}^{-1}=\frac{1}{\cos\phi_{x}^{g_{t}}}\cdot \left[\begin{array}{ccc} \cos\phi_{y}^{g_{t}}\cos\phi_{x}^{g_{t}} & 0 & \sin\phi_{y}^{g_{t}}\cos\phi_{x}^{g_{t}}\\ \sin\phi_{y}^{g_{t}}\sin\phi_{x}^{g_{t}} & \cos\phi_{y}^{g_{t}} & -\cos\phi_{y}^{g_{t}}\sin\phi_{x}^{g_{t}}\\ -\sin\phi_{y}^{g_{t}} & 0 & \cos\phi_{y}^{g_{t}} \end{array}\right],$$

The dynamics model of the conventional initial alignment algorithm with a large misalignment angle proposed for the non-polar region is chosen as the comparison model in this paper. The dynamics model of the conventional initial alignment algorithm with a large misalignment angle can be described as Equation (39).

(39) $$\{\begin{array}{l} {\dot{\phi }}^{g}=C_{g}{}^{-1}\left[\left(I-C_{g}^{g^{\prime }}\right)\omega_{ig}^{g}+C_{\omega egv}\delta V^{g}-C_{b}^{g^{\prime }}\varepsilon^{b}\right]\\ \delta {\dot{V}}^{g}=\left[I-(C_{g}^{g^{\prime }}{)}^{T}\right]C_{b}^{g}f^{b}+\left[V^{g}\times C_{\omega egv}-\left(2\omega_{ie}^{g}+\omega_{eg}^{g}\right)\times \right]\cdot \delta V^{g}+C_{b}^{g^{\prime }}\nabla^{b}\\ {\dot{\varepsilon }}^{b}=0\\ {\dot{\nabla }}^{b}=0 \end{array},$$

where $C_{g}{}^{-1}$ can be described as:

(40) $$C_{g}{}^{-1}=\frac{1}{\cos\phi_{x}^{g}}\cdot \left[\begin{array}{ccc} \cos\phi_{y}^{g}\cos\phi_{x}^{g} & 0 & \sin\phi_{y}^{g}\cos\phi_{x}^{g}\\ \sin\phi_{y}^{g}\sin\phi_{x}^{g} & \cos\phi_{y}^{g} & -\cos\phi_{y}^{g}\sin\phi_{x}^{g}\\ -\sin\phi_{y}^{g} & 0 & \cos\phi_{y}^{g} \end{array}\right],$$

### 4.2. Observation Model

The velocity errors are chosen as the observation states in the polar transversal initial alignment algorithm. The observation states and the observation model of the polar transversal initial alignment algorithm with a large misalignment angle can be described as Equations (41) and (42). The UUV is temporary anchored. The ideal velocity of UUV is zero. Therefore, the measurement results from the Doppler velocity log (DVL) can be used as the velocity errors. The velocity errors are the observation states.

(41) $$Z=\delta V^{g_{t}},$$

(42) Z=HX+V,

where H is the observation matrix, and V is the measurement noise vector which is the independent Gaussian white noise $V_{k}∼N\left(0,R_{k}\right)$ and R is the measurement noise covariance.

(43) $$H={\left[0_{2\times 3}\;I_{2\times 2}\;0_{2\times 3}\;0_{2\times 3}\right]}^{T},$$

### 4.3. Observability Analysis

It is relatively easy to analyze the observability of a steady system [28]. However, the initial alignment algorithm for UUVs in polar regions is a time-varying system. There is a swing in the initial alignment process. The Piece-Wise Constant System (PWCS) observability analysis algorithm was proposed by Goshen-Meskin and Bar-Itzhack [29] to realize the observability analysis of time-varying systems. Based on this algorithm, a Singular Value Decomposition (SVD) observability analysis algorithm was proposed by Cheng [30,31,32]. The SVD observability analysis algorithm is employed in this paper to realize the observability analysis of the proposed algorithm. The PWCS model can be described as Equation (44).

(44) $$\left\{ \begin{array}{l} \dot{X}(t_{j})=A_{j}X(t_{j})+B_{j}W(t_{j})\\ Z_{j}(t_{j})=H_{j}X(t_{j})+V(t_{j}) \end{array}\right.,$$

where j=1,2,…,r represent the period of time. The observability matrix in *j* time period can be described as ${\tilde{Q}}_{j}$. The Total Observability Matrix (TOM) can be described as $\tilde{Q}(r)$. The Stripped Observability Matrix (SOM) can be described as ${\tilde{Q}}_{s}(r)$. The calculation of the TOM is complex. Therefore, SOM is widely used.

(45) $${\tilde{Q}}_{j}=\left[\begin{array}{ccccc} {\left(H_{j}\right)}^{T} & {\left(H_{j}A_{j}\right)}^{T} & {\left(H_{j}A_{j}^{2}\right)}^{T} & \cdots & {\left(H_{j}A_{j}^{n-1}\right)}^{T} \end{array}\right],$$

(46) $$\tilde{Q}(r)={\left[\begin{array}{cccc} {\left({\tilde{Q}}_{1}\right)}^{T} & {\left({\tilde{Q}}_{2}e^{A_{1}\Delta_{1}}\right)}^{T} & \cdots & {\left({\tilde{Q}}_{r}e^{A_{r-1}\Delta_{r-1}}\cdots e^{A_{1}\Delta_{1}}\right)}^{T} \end{array}\right]}^{T},$$

(47) $${\tilde{Q}}_{s}(r)={\left[\begin{array}{cccc} {\left({\tilde{Q}}_{1}\right)}^{T} & {\left({\tilde{Q}}_{2}\right)}^{T} & \cdots & {\left({\tilde{Q}}_{r}\right)}^{T} \end{array}\right]}^{T},$$

The singular value decomposition of the SOM can be described as Equation (48).

(48) $${\tilde{Q}}_{s}(r)=U\Sigma V^{H},$$

where

(49) $$\left\{ \begin{array}{l} U=\left[\begin{array}{cccc} u_{1} & u_{2} & \cdots & u_{m} \end{array}\right]\\ V=\left[\begin{array}{cccc} v_{1} & v_{2} & \cdots & v_{m} \end{array}\right]\\ \Sigma ={\left[\begin{array}{cc} S & O_{(m-r)\times r} \end{array}\right]}^{T}\\ S=diag(\sigma_{1},\cdots ,\sigma_{r}) \end{array}\right.,$$

Therefore,

(50) $$Z={\tilde{Q}}_{s}(r)X(0),$$

(51) $$Z=\sum_{i=1}^{r}\sigma_{i}\{v_{i}^{T}X(0)\}u_{i},$$

(52) $$X(0)={\left(U\Sigma V^{H}\right)}^{-1}Z=\sum_{i=1}^{r}\frac{u_{i}^{T}X(0)v_{i}}{\sigma_{i}},$$

The observability of the system can be defined as Equation (53).

(53) $$\eta_{k}=\frac{\sigma_{m}}{\sigma_{0}},m=1,2,\cdots ,n,$$

where, $\eta_{k}$ is the observability of the *k*th state. In addition, $\sigma_{0}$ is the singular value of the observation state. In addition, $\sigma_{m}$ is the singular value when the ${\left[u_{i}^{T}Zv_{i}/\sigma_{m}\right]}_{k}$ is max.

The observability analysis of the system can be described as Table 1.

As shown in Table 1, the observability degree of $\delta V_{x}$ and $\delta V_{x}$ are 1.0. Because $\delta V_{x}$ and $\delta V_{x}$ are also the observation states, this means that they are observable. The observability degree can reflect the accuracy and the convergence time of the state estimate. Some of the observability degrees are low. This does not mean that the state cannot be estimated. Rather, it indicates that the estimation accuracy of the states will be low, or that the convergence time is long [32]. For example, $\phi_{z}$ has lower accuracy and longer convergence time than other states.

### 4.4. Filter Algorithm

According to the assumption of a large misalignment angle, nonlinear models are deduced. Therefore, the conventional KF will be invalid for the data fusion. Although EKF can be used for the data fusion of nonlinear models, the calculation of the Jacobi matrix is complex. The higher-order terms are neglected by the EKF. In addition, ignoring the higher-order terms will result in inaccurate results. For a nonlinear system, approximating a statistical distribution is easier than approximating a nonlinear transformation. Based on this idea, Unscented Transformation (UT) and UKF were proposed by Julier and Uhlmann [33,34]. UKF is more accurate and convenient than EKF for initial alignment algorithms with a large misalignment angle [33,34]. Therefore, the UKF is chosen to realize the data fusion in this paper. In the conventional UKF, the states need to be extended. However, when the observation noises are additive noises, there is no need to extend the states. In this paper, the observation noises are additive noises [35]. Therefore, a simplified UKF is chosen to fuse the data. The simplified UKF can be described as Equations (54)–(67).

(54) $${\hat{x}}_{0}=E\left[x_{0}\right],$$

(55) $$P_{0}=E\left[(x_{0}-{\hat{x}}_{0}){\left(x_{0}-{\hat{x}}_{0}\right)}^{T}\right],$$

(56) $$\chi_{i,k-1}=\left\{ \begin{array}{ll} {\hat{x}}_{k-1} & i=0\\ {\hat{x}}_{k-1}+(\sqrt{(n+\kappa )P_{k-1}}), & i=1,2,\cdots ,n\\ {\hat{x}}_{k-1}-(\sqrt{(n+\kappa )P_{k-1}}), & i=n+1,\cdots ,2n \end{array}\right.,$$

(57) $$W_{i}=\left\{ \begin{array}{ll} \kappa /(n+\kappa ),\; & i=0\\ 1/2(n+\kappa ), & i=1,2,\cdots ,2n \end{array}\right.,$$

(58) $$X_{i,k/k-1}=f(\chi_{i,k-1}),$$

(59) $${\hat{x}}_{k/k-1}=\sum_{i=0}^{2n}W_{i}X_{i,k/k-1},$$

(60) $$P_{k/k-1}=\sum_{i=0}^{2L}W_{i}(X_{i,k/k-1}-{\hat{x}}_{k/k-1}){\left(X_{i,k/k-1}-{\hat{x}}_{k/k-1}\right)}^{T}+Q_{k-1},$$

(61) $$Z_{i,k/k-1}=H_{k}\chi_{i,k/k-1},$$

(62) $${\hat{𝓏}}_{k/k-1}=H_{k}{\hat{x}}_{k/k-1},$$

(63) $${\hat{x}}_{k}={\hat{x}}_{k/k-1}+K_{k}({𝓏}_{k}-{\hat{𝓏}}_{k/k-1}),$$

(64) $$K_{k}=P_{xz,k}P_{z,k}^{-1},$$

(65) $$P_{k}=P_{k/k-1}-K_{k}P_{z,k}K_{k}{}^{T},$$

(66) $$P_{xz,k}=\sum_{i=0}^{2n}W_{i}(X_{i,k/k-1}-{\hat{x}}_{k/k-1}){\left(Z_{i,k/k-1}-{\hat{𝓏}}_{k/k-1}\right)}^{T},$$

(67) $$P_{z,k}=H_{k}P_{k/k-1}H_{k}{}^{T}+R_{k},$$

## 5. Results and Discussion

### 5.1. Simulation Results

To imitate the polar region, the initial conditions of the simulation are set as follows. The latitude and the longitude of the initial location are set as 85° and 126°, respectively. The simulation time and the filter period are 600 s and 0.1 s, respectively. The gyro constant drifts and the gyro random drifts are set as 0.02°/h and 0.001°/h, respectively. The accelerometer constant bias and the accelerometer random bias are $1\times 10^{-4}g_{0}$ and $1\times 10^{-5}g_{0}$, respectively. The simplified UKF is used to realize the data fusion. The initial values of the state estimation covariance $P_{0}$, the system noise covariance Q, and the measurement noise covariance R are set as follows.

$$P_{0}=diag\left\{\begin{array}{l} {\left(10{}^{\circ}\right)}^{2},{\left(20{}^{\circ}\right)}^{2},{\left(60{}^{\circ}\right)}^{2},{\left(0.1\;m/s\right)}^{2},{\left(0.1\;m/s\right)}^{2},{\left(0.02\pi /180/3600\;rad/s\right)}^{2},\\ {\left(0.02\pi /180/3600\;rad/s\right)}^{2},{\left(0.02\pi /180/3600\;rad/s\right)}^{2},\\ {\left(1\times 10^{-4}g_{0}\;m/s^{2}\right)}^{2},{\left(1\times 10^{-4}g_{0}\;m/s^{2}\right)}^{2} \end{array}\right\}$$

$$Q=\mathrm{diag}\left\{\begin{array}{l} {\left(0.001\pi /180/3600\;rad/s\right)}^{2},{\left(0.001\pi /180/3600\;rad/s\right)}^{2},\\ {\left(0.001\pi /180/3600\;rad/s\right)}^{2},{\left(1\times 10^{-5}g_{0}\;m/s^{2}\right)}^{2},{\left(1\times 10^{-5}g_{0}\;m/s^{2}\right)}^{2} \end{array}\right\}$$

$$R=diag\left\{{\left(0.01\;m/s\right)}^{2},{\left(0.01\;m/s\right)}^{2}\right\}$$

**Figure 2:**
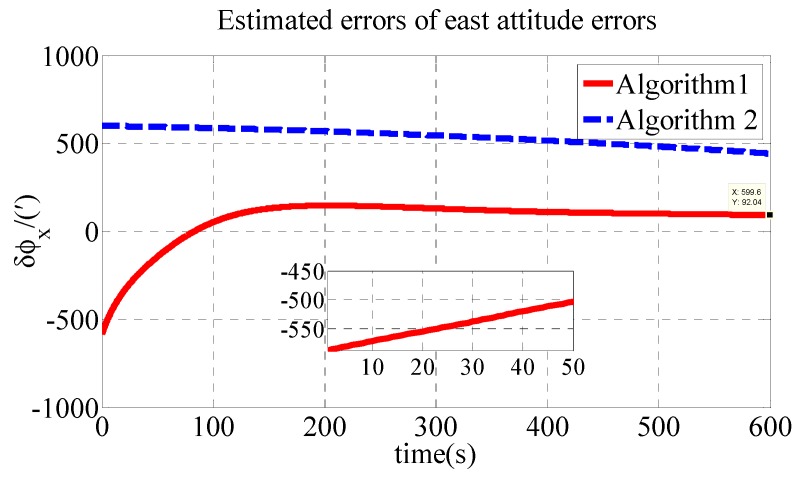
Estimated errors of east attitude errors in simulation.

**Figure 3:**
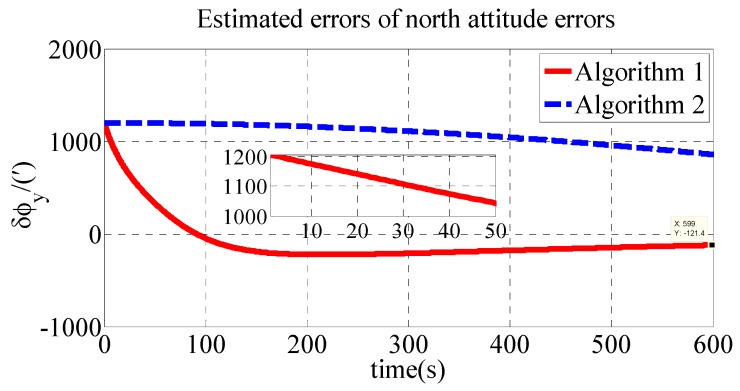
Estimated errors of north attitude errors in simulation.

**Figure 4:**
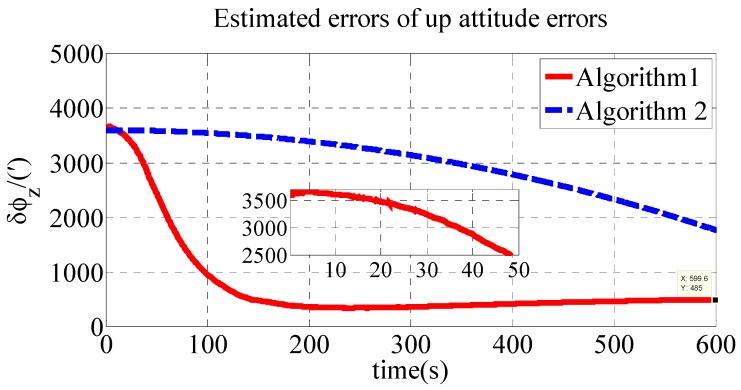
Estimate errors of up attitude errors in simulation.

The polar transversal initial alignment algorithm for UUVs with large misalignment angles proposed in this paper is called Algorithm 1. The conventional initial alignment algorithm for UUVs with large misalignment angles is called Algorithm 2. In Algorithm 2, the geographic frame is chosen as the navigation frame, as shown in Equations (39) and (40) [35]. Algorithm 1 is compared with Algorithm 2 in the polar region to verify the effectiveness of the proposed algorithm in the polar region. The estimated errors of the attitude errors in the simulation are shown in Figure 2, Figure 3 and Figure 4. The results of the initial alignment algorithms are shown in Table 2.

As shown in Table 2, the initial alignment results of $\phi_{x}$, $\phi_{y}$ and $\phi_{z}$ in Algorithm 1 are 92.04′, −121.40′ and 485′, respectively. The initial alignment results of $\phi_{x}$, $\phi_{y}$ and $\phi_{z}$ in Algorithm 2 are 442.6′, 857.7′ and 1769′, respectively. The simulation results demonstrate that the proposed polar transversal initial alignment algorithm has better performance than the conventional initial alignment algorithm in the polar region. The attitude errors converge to near zero quickly. In addition, the attitude errors are then stable near zero. The polar transversal initial alignment algorithm can realize the initial alignment in the polar region effectively, especially when large misalignment angles occur.

**Figure 5:**
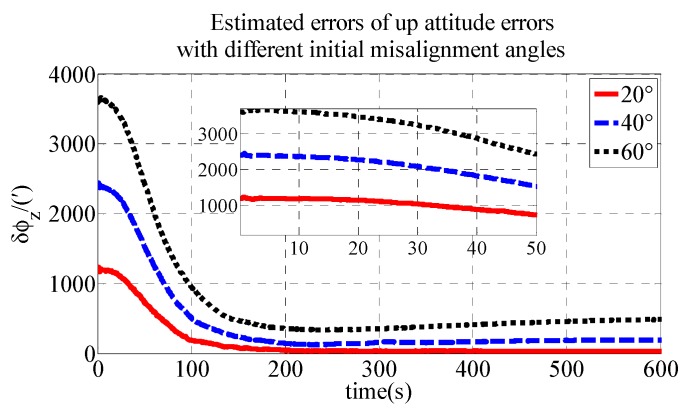
Estimated errors of up attitude errors with different misalignment angles in simulation.

To express the influence of different initial misalignment angles, simulations are performed with different misalignment angles in the polar region. Take the up direction as an example; different misalignment angles are compared in the simulation. Detailed simulation results are presented in Figure 5.

As shown in Figure 5, the estimated errors of up attitude errors converge quickly within 100 s. In addition, the estimated errors are then stable near zero. Smaller misalignment angles have faster initial alignment speeds and better initial alignment accuracy.

### 5.2. Experiment Results

**Figure 6:**
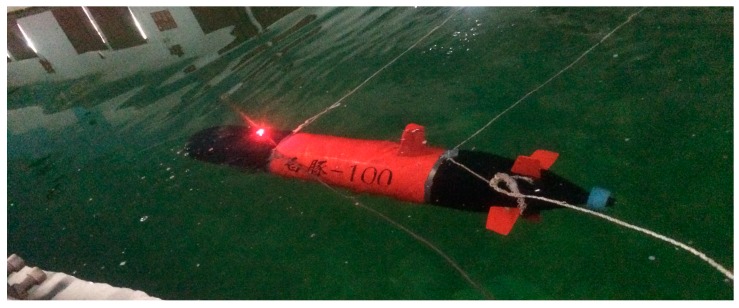
White Dolphin-100 UUV.

The experiment was performed in the form of a semi-physical simulation. This is because of the geography restrictions of the authors’ country. The experiment was performed with the White Dolphin-100 UUV, shown in Figure 6. In the semi-physical simulation, the experimental data were measured in the non-polar region. The simulation was performed with these data. Both the simulation data and the experimental data together comprised the semi-physical data. The semi-physical data included the special force ${\hat{f}}^{b}$ and the angular velocity ${\hat{\omega }}_{ib}^{b}$. The special force ${\hat{f}}^{b}$ is composed of the true value of the special force $f^{b}$ and the accelerometer drifts $\delta f^{b}$. The angular velocity ${\hat{\omega }}_{ib}^{b}$ is composed of the true value of the angular velocity $\omega_{ib}^{b}$ and the gyro drifts $\delta \omega_{ib}^{b}$. The true value of the special force $f^{b}$ and the angular velocity $\omega_{ib}^{b}$ are related to the sailing mission. Therefore, they can be obtained from the simulation. The accelerometer drifts $\delta f^{b}$ and the gyro drifts $\delta \omega_{ib}^{b}$ are related to the accelerometer and the gyro. Therefore, they can be extracted from the practical measured data [36]. As shown in Equation (68), both the simulation data and the experimental data together comprised the semi-physical data.

(68) $$\{\begin{array}{l} {\hat{f}}^{b}=f^{b}+\delta f^{b}\\ {\hat{\omega }}_{ib}^{b}=\omega_{ib}^{b}+\delta \omega_{ib}^{b} \end{array},$$

The experimental setup can be described as follows. Due to the geographical restrictions, the experiment is conducted in the non-polar region. The practical measured data is composed of the gyro drifts and the accelerometer bias, which can be obtained from the experiment [36]. They are obtained from an experiment conducted in a rectangular pool. The location of the pool is (45°73′ N 127°41′ E). The experiment lasts for 3600 s. The sampling frequency is 100 Hz. The pitch angle, the roll angle and the yaw angle of the UUV are described by the sine function. The amplitude of these three angles is 4°, 5° and 3°, respectively. The period of these three angles is 3 s, 5 s, and 7 s, respectively. The initial phase of these three angles is 0°, 0° and 0°, respectively. The UUV used for the experiment is built by our laboratory. This UUV is called the White-Dolphin-100 UUV. This UUV is equipped with a depth sensor, inertial measurement unit (IMU), OCTANS, DVL, underwater camera, Global Positioning System (GPS), and other sensors. Among these sensors, the IMU plays the most important role in this experiment. In addition, the initial position is provided by GPS. The results of the practical measured data can be described as Table 3.

The semi-physical simulation setup can be described as follows. The initial position is set as follows. The latitude and the longitude of the initial location are set as 85° and 126°, respectively. The experimental data, including the accelerometer bias and the gyro drifts, are as follows. The accelerometer random biases are $0.00156\;m/s^{2}$, $0.001747\;m/s^{2}$, and $0.0004063\;m/s^{2}$, respectively. The gyro constant drift is 0.02°/h. The gyro random drifts are $4.094\times 10^{-6}\;rad/s$, $4.308\times 10^{-6}\;rad/s$, and $2.386\times 10^{-6}\;rad/s$, respectively. The simplified UKF is used to realize the data fusion. Therefore, the initial values of the state estimation covariance $P_{0}$, the system noise covariance Q, and the measurement noise covariance R are set as follows.

$$P_{0}=diag\left\{\begin{array}{l} {\left(10{}^{\circ}\right)}^{2},{\left(20{}^{\circ}\right)}^{2},{\left(60{}^{\circ}\right)}^{2},{\left(0.1\;m/s\right)}^{2},{\left(0.1\;m/s\right)}^{2},{\left(0.02\pi /180/3600\;rad/s\right)}^{2},\\ {\left(0.02\pi /180/3600\;rad/s\right)}^{2},{\left(0.02\pi /180/3600\;rad/s\right)}^{2},\\ {\left(1\times 10^{-4}g_{0}\;m/s^{2}\right)}^{2},{\left(1\times 10^{-4}g_{0}\;m/s^{2}\right)}^{2} \end{array}\right\}$$

$$Q=\mathrm{diag}\left\{\begin{array}{l} {\left(4.094\times 10^{-6}\;rad/s\right)}^{2},{\left(4.308\times 10^{-6}\;rad/s\right)}^{2},\\ {\left(2.386\times 10^{-6}\;rad/s\right)}^{2},{\left(0.00156\;m/s^{2}\right)}^{2},\;{\left(0.001747\;m/s^{2}\right)}^{2} \end{array}\right\}$$

$$R=diag\left\{{\left(0.01\;m/s\right)}^{2},{\left(0.01\;m/s\right)}^{2}\right\}$$

**Figure 7:**
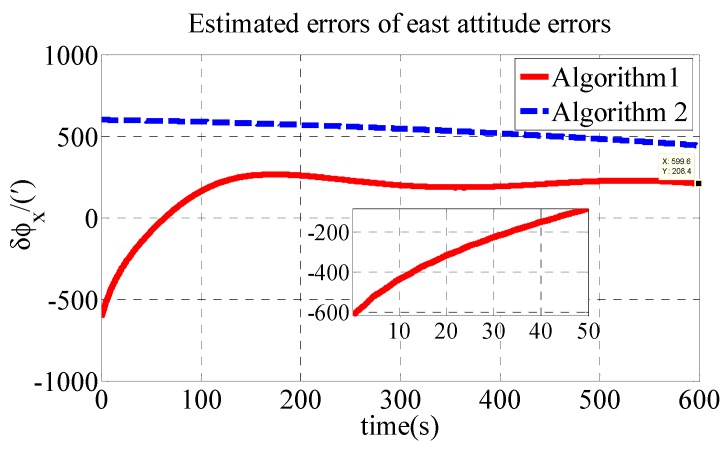
Estimated errors of east attitude errors in experiment.

**Figure 8:**
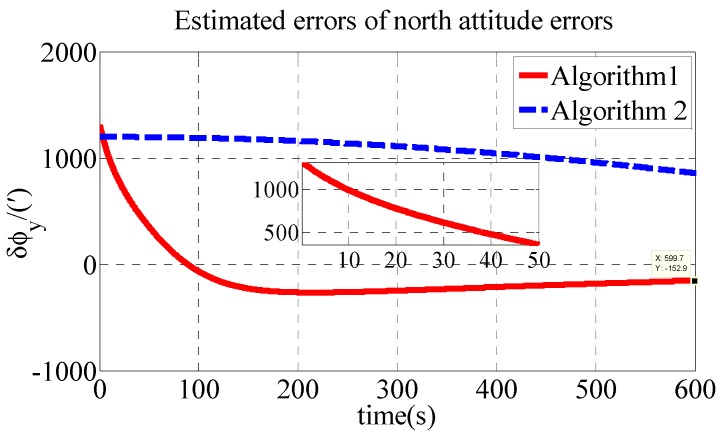
Estimated errors of north attitude errors in experiment.

**Figure 9:**
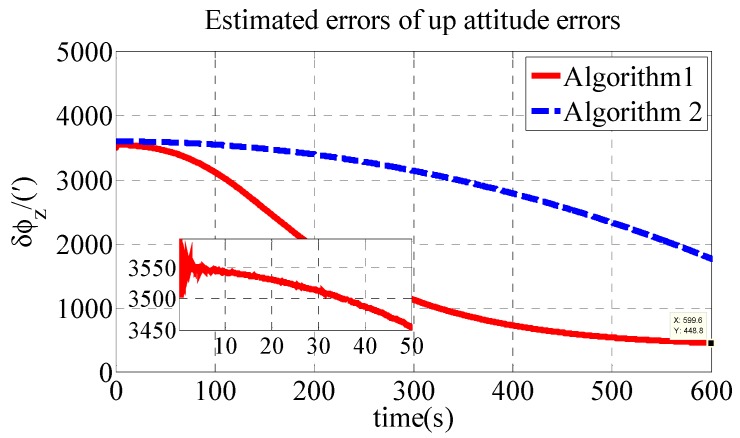
Estimate errors of up attitude errors in experiment.

The other parameters are the same as those in the simulation. The estimated errors of attitude errors in the experiment are shown in Figure 7, Figure 8 and Figure 9.

As shown in Table 4, the initial alignment results of $\phi_{x}$, $\phi_{y}$ and $\phi_{z}$ in Algorithm 1 are 208.4′, −152.9′ and 448.8′, respectively. The initial alignment results of $\phi_{x}$, $\phi_{y}$ and $\phi_{z}$ in Algorithm 2 are 442.7′, 858′ and 1773′, respectively. The experiment results are similar to those of the simulation. The proposed polar transversal initial alignment algorithm exhibits better performance than that of the conventional initial alignment algorithm for UUV in the polar region. The errors converge quickly. In addition, after the convergence, the errors are stable near zero.

### 5.3. Discussion

SINSs are widely used in the navigation of UUVs because of their autonomy and concealment. Based on the choice of navigation frame, SINSs can be divided into SINSs based on the north-oriented frame, SINSs based on the earth-centered earth-fixed (ECEF) frame, and so on. In SINSs based on the north-oriented frame, the geographic frame is chosen as the navigation frame. Based on the characteristics of UUVs, SINSs based on the north-oriented frame are the most widely used in UUVs. UUVs sail near the surface of the earth. Using the north-oriented frame, the output component of the accelerometer in the SINS is not affected by gravitational acceleration. In addition, the attitude output directly reflects the posture of the carrier. There is no need to convert the coordinate system. The volume of calculation is reduced. Therefore, SINSs based on the north-oriented frame are widely used in UUVs [17]. According to the analysis above, the conventional algorithm described in this paper refers to the initial alignment algorithm in SINS based on the north-oriented frames. Although SINSs based on north-oriented frames are suitable for UUVs, there are problems in these algorithms when using them in the polar region. The problems can be described as follows [14]. Therefore, the transversal initial alignment algorithm is proposed in this paper.

In the polar region, the Earth’s meridians converge quickly. In addition, the latitude approaches 90° in the polar region. These cause inaccuracy and calculation overflow in conventional algorithms when applied in the polar region. Therefore, conventional algorithms are invalid in the polar region. In the conventional algorithms, the instruction angular velocity can be described as Equation (69), and the up-directional instruction angular velocity error can be expressed as Equation (70).

(69) $$\omega_{en}^{n}=\left[\begin{array}{c} \omega_{enE}^{n}\\ \omega_{enN}^{n}\\ \omega_{enU}^{n} \end{array}\right]=\left[\begin{array}{c} -v_{N}/R_{Mh}\\ v_{E}/R_{Nh}\\ \left(v_{E}/R_{Nh}\right)\tan L \end{array}\right],$$

(70) $$\delta \omega_{enU}^{n}=\left(\delta v_{E}/R_{Nh}\right)\tan L,$$

Based on Equation (70), the up-directional instruction angular velocity error is related to the tangent value of the latitude. Therefore, the tangent value of the latitude approaches infinity when the UUV approaches the polar region. The up-directional instruction angular velocity error approaches infinity in the polar region. This causes inaccuracy and calculation overflow in conventional algorithms when applied in the polar region. To solve these problems, the transversal algorithm is introduced in this paper.

To facilitate comparative discussion, the conventional initial alignment algorithm proposed for UUVs in non-polar regions is chosen as the comparison model. This is based on the conventional north-oriented SINS algorithm. The performance of these two algorithms is compared in the polar region. The simulation and the experiment results demonstrate that the polar transversal initial alignment algorithm is superior to the conventional initial alignment algorithm for UUVs in the polar region. Compared with the conventional initial alignment algorithm, the polar transversal initial alignment algorithm exhibited more accuracy. The advantages of the polar transversal initial alignment algorithm for UUV with a large misalignment angle are concluded as follows.

The conventional initial alignment algorithms have difficulties in the polar region, mainly in the following two aspects. First, in the conventional initial alignment algorithm, the error of the up-directional angular component in the command angular velocity is related to the tangent value of the latitude. In the polar region, the latitude tends to 90°, and the tangent value tends to infinity. Therefore, the error of the up-directional angular component in the command angular velocity tends to infinity in the polar region. Second, due to the rapid convergence of the Earth meridians in the polar region, very small longitude errors will result in large positional deviation when applying the conventional latitude and longitude expression in the polar region. The conventional position expression is unsuitable for the polar region. Therefore, the conventional initial alignment algorithm is unsuitable for the polar region. As the simulation and the experiment results demonstrate, the initial alignment accuracy of conventional initial alignment algorithms does not satisfy the requirements.

The polar transversal initial alignment algorithm overcomes the above problems by rotating the frames 90° around the y axis of the earth-centered earth-fixed frame. Therefore, the polar region in the conventional initial alignment algorithm is equivalent to the equatorial region in the polar transversal initial alignment algorithm. In the polar region, the pseudo latitude tends to zero and the error of the upside angular component in the command angular velocity tends to zero. In addition, the slow convergence of the pseudo meridians have no impact on the position expression. Furthermore, large misalignment angles are considered in this paper, and non-linear models are established. The simplified UKF realizes the data fusion. This is more suitable for the complex environment in the polar region. In the second comparison simulation, different misalignment angles are compared. The simulation results demonstrate that smaller misalignment angles have faster initial alignment speeds and better initial alignment accuracies.

Based on the analysis above, the polar transversal initial alignment algorithm with a large misalignment angle proposed in this paper has better performance than the conventional initial alignment algorithm with a large misalignment angle in the polar region. The polar transversal initial alignment algorithm for UUVs with a large misalignment angle is effectively suited for the initial alignment in the polar region.

## 6. Conclusions

In this paper, transversal frames are established by rotating the frames 90° around the y axis of the e frame. Based on the transversal frames, the polar transversal initial alignment algorithm is proposed for UUV with a large misalignment angle in the polar region. The transversal geographic frame is chosen as the navigation frame in this algorithm. In the transversal frames, the polar region is equivalent to the equatorial region. Therefore, the rapid convergence of the Earth meridians has no impact on the polar transversal initial alignment algorithm. Two comparison simulations and an experiment are performed to verify the effectiveness of the proposed polar transversal initial alignment algorithm. The simulation and experiment results demonstrate that the polar transversal initial alignment algorithm for UUVs can realize accurate initial alignment in the polar region, especially when large misalignment angles occur. This algorithm is suitable for the initial alignment of UUVs with a large misalignment angle in the polar region.

## Figures and Tables

**Table 1:** Observability analysis of the system.

**Parameters**	$\phi_{x}$	$\phi_{y}$	$\phi_{z}$	$\delta V_{x}$	$\delta V_{y}$	$\varepsilon_{x}$	$\varepsilon_{y}$	$\varepsilon_{z}$	$\nabla_{x}$	$\nabla_{y}$	$\nabla_{z}$
**Observability Degree**	9.9	9.9	<10^−15^	1.0	1.0	9.8	9.8	6.97 × 10^−5^	<10^−15^	<10^−15^	<10^−15^

**Table 2:** Attitude errors after initial alignment in the simulation.

Parameters	Algorithm 1	Algorithm 2
$\phi_{x}$/(′)	92.04	442.6
$\phi_{y}$/(′)	−121.4	857.7
$\phi_{z}$/(′)	485	1769

**Table 3:** The practical measured data.

Parameters	Value
Gyro constant drifts	0.02°/h
Gyro random drifts	4.094 × 10^−6^ rad/s
	4.308 × 10^−6^ rad/s
	2.386 × 10^−6^ rad/s
Accelerometer constant	1 × 10^−4^ g_0_ m/s^2^
Accelerometer random bias	0.00156 m/s^2^
	0.001747 m/s^2^
	0.0004063 m/s^2^

**Table 4:** Attitude errors after initial alignment in the experiment.

Parameters	Algorithm 1	Algorithm 2
$\phi_{x}$/(′)	208.4	442.7
$\phi_{y}$/(′)	−152.9	858
$\phi_{z}$/(′)	448.8	1773
